# Long non-coding RNA ANRIL-mediated inflammation response is involved in protective effect of rhein in uric acid nephropathy rats

**DOI:** 10.1186/s13578-019-0273-3

**Published:** 2019-01-17

**Authors:** Jiacai Hu, Daochun Wang, Hao Wu, Zhijie Yang, Na Yang, Junjun Dong

**Affiliations:** 1 Department of Traditional Chinese Medicine, Renmin Hospital of Wuhan University, No. 99 Zhangzhidong Road, Wuhan, 430060 China; 2 Department of Acupuncture and Moxibustion, Renmin Hospital of Wuhan University, Wuhan, 430060 China

**Keywords:** Long non-coding RNA ANRIL, UAN, Inflammatory response, Rhein, Hyperuricemia

## Abstract

**Background:**

The aim of this study was to investigate the role of long non-coding RNAs (LncRNAs) antisense non-coding RNA in the INK4 locus (ANRIL) in anti-inflammation of rhein in uric acid nephropathy (UAN) rats.

**Methods:**

Rat models of UAN were induced by adenine and potassium oxonate. Enzyme-linked immunosorbent assay (ELISA) was performed to assess inflammation factor in serum and supernatant. ANRIL mRNA level was detected using real-time reverse transcription PCR (qRT-PCR). Immunostaining was used to observe pathological changes of renal tissues in rats.

**Results:**

ANRIL and inflammatory factor levels were highly expressed in patient with UAN. Furthermore, rhein showed an observable effect on anti-inflammatory and renal protection in UAN rats, rhein inhibited expressions of ANRIL in vivo or in vitro. Besides, ANRIL-mediated inflammatory response attenuated protective effect of rhein.

**Conclusions:**

ANRIL-mediated inflammatory response attenuated the protective effect of rhein in UAN rats. This study showed an understanding of the role and mechanism of ANRIL in UAN, which provides a new target and therapy for the prevention and treatment of UAN.

**Electronic supplementary material:**

The online version of this article (10.1186/s13578-019-0273-3) contains supplementary material, which is available to authorized users.

## Background

Hyperuricemia results from overproduction or insufficient excretion of uric acid, which is characterised by uric acid crystals to a tubular obstruction [1]. Serum urate levels at a consistently high concentration can cause urate crystal deposition in ducts and/or in the interstitium, and stimulates a series of inflammatory responses to further develop kidney disease [2, 3], named Uric acid nephropathy (UAN). The anti-hyperuricemic and anti-inflammatory actions are the key point in conservancy of renal function. Many studies have indicated that traditional herbal medicines play an important role in nephroprotection [4–8]. Rhein, an active anthraquinone compound, is extracted from *Rheum palmatum* L., *Aloe barbadensis* Miller, *Cassia angustifolia* Vahl., and *Polygonum multiflorum* Thunb [9]. Rhein is involved in various pharmacological activities, such as anti-inflammatory, antioxidative, antitumor and purgative effects [5, 10]. Rhein was also found to decrease the development of the inflammatory in joint diseases [11]. Additionally, it has been reported that rhein significantly inhibited the production of IL-1β, TNF-α and improved kidney function of the UAN mice [5].

Long non-coding RNAs (LncRNAs) are defined as non-protein coding transcripts, with a length more than 200 nucleotides. Accumulating evidence showed that the lncRNAs are considered as critical gene regulator in biological processes of multiple disease, including cell growth and differentiation, development and inflammation [12–14]. Long non-coding RNA ANRIL (CDKN2B antisense RNA 1) in the INK4 locus is transcribed as a 3.8-kb-long RNA in the opposite direction of the INK4B-ARFINK4A gene cluster [15]. Recently, it has been reported that ANRIL gene is associated with cancers, coronary disease, intracranial aneurysm and type 2 diabetes in common disease genomewide association studies [16]. Many previous studies reveal that ANRIL is up-regulated in hepatocellular carcinoma [13], bladder cancer [17], gastric cancer [18], prostate cancer [19], ovarian cancer [20] and lung cancer [21]. Additionally, research demonstrates that ANRIL controlled by NF-κB has a key role to regulate a subset of pro-inflammatory genes [22]. However, the function of ANRIL associated with inflammatory response in UAN remains largely unrevealed.

Therefore, the aim of this study was to examine whether ANRIL-regulated inflammatory response plays a role in protective effect of rhein on UAN. In the present study, we used an experimental model of UAN induced by adenine and potassium oxonate in rat to explore the mechanism of ANRIL in UAN rats. In our study, we focused mainly on the effect of ANRIL-mediated inflammatory in UAN rats.

## Methods

### Clinical specimens

The blood samples were collected from patients with UAN (n = 25) and age-matched healthy volunteers (n = 25). This study was approved by the Ethics Committee of Renmin Hospital of Wuhan University and all patients provided informed consent.

### Animal model of UAN and drug administration

Male Sprague–Dawley (SD) rats were purchased from Experimental Animal Center of Shanghai (Shanghai, China). All experimental procedures were carried out in accordance with the guidelines for the Care and Use of Laboratory Animals of the National Institutes of Health. The model of adenine-induced hyperuricemia in rats (n = 60) was established according to our previous research [8], 12 rats were fed with normal chow as control (control group) and all animal had free access to water. After 20 days, model rats returned to the normal diet. Model rats were randomly divided into five groups (12 animals were used for each group, n = 12) and all rats were treated as follows: control group and model group (n = 12), in which rats underwent gastric perfusion of 4 mL distilled water; Rhein-L group (n = 12), in which rats underwent gastric perfusion of 75 mg/kg rhein; Rhein-M group (n = 12), in which rats underwent gastric perfusion of 150 mg/kg rhein; Rhein-H group (n = 12), in which rats underwent gastric perfusion of 300 mg/kg rhein; allopurinol group (n = 12), in which rats underwent gastric perfusion of 10 mg/kg allopurinol. Treatment was continued for 14 days. Each rat was sacrificed under anaesthesia by intraperitoneal injection of sodium pentobarbital (50 mg/kg). Blood samples were collected by carotid artery intubation and centrifuged at 3000×*g*, 4 °C for 5 min to get the serum. The bilateral kidneys were quickly dissected and stored at − 80 °C after weighing until follow-up experiment analysis.

### Cell culture and treatment

Peripheral blood mononuclear cells (PBMCs) were isolated from peripheral blood of patients with UAN and healthy volunteers by lymphocyte separation liquid (Tianjin Haoyang Biotech Company, Tianjin, China). Normal rat kidney epithelial cell line NRK-52E was purchased from the American Type Culture Collection (ATCC, USA), cultivated in complete Dulbecco’s modified Eagle’s medium (DMEM, Gibco BRL, USA) supplemented with 10% fetal bovine serum (FBS, Gibco BRL, USA). The cells were maintained at 37 °C with 5% CO_2_. NRK-52E cells treated with or without TNF-α (25 ng/mL)/IL-1β (10 ng/mL) (Peprotech) for 24 h, or cells were treated with rhein at 10, 20, 40 μg/mL for 2 h, then added TNF-α, next co-cultured 24 h and collected.

### Cells transfection

NRK-52E cells were seeded in six-well plates and transfected with ANRIL gene overexpression vector or pcDNA3.1 (Guangzhou Ruibo Biotechnology Co. LTD., Guangzhou, China) using lipofectamine 2000 transfection reagent (Invitrogen, Carlsbad, CA, USA) according to the manufacture’s protocol. Then cells were treated with TNF-α and different concentration of rhein.

### Measurement of serum indexes of kidney function

Serum uric acid (Sur), serum creatinine (Scr), blood urea nitrogen (BUN), 24 h-proteinuria and serum β2-MG levels in rats were measured according to the previous description [8].

### Enzyme-linked immunosorbent assay (ELISA)

The levels of TNF-α, IL-1β, IL-6 and IL-8 in serum from patients with UAN and healthy volunteers, UAN rat and control or cells culture medium were measured with an ELISA kit (R&D Systems, USA) flowing the manufacturer’s protocol.

### Western blot

Total protein was separated using Radio-Immunoprecipitation Assay (RIPA) buffer (Santa Cruz Biotechnology, Santa Cruz, CA, USA). Total protein extract was incubated with 12% sodium dodecyl sulfate polyacrylamide gel electrophoresis (SDS-PAGE), transferred to polyvinylidene fluoride (PVDF) membranes and blocked with 5% skim milk at room temperature for 1 h. The membranes were incubated with primary antibodies against NF-kB p65, phospho-NF-kB p65 (1:1000; Cell Signaling Technology, Danvers, MA, USA) at 4 °C overnight, then incubated with horseradish peroxidase-conjugated goat anti-rabbit secondary antibodies (Cell Signaling Technology) at room temperature for 2 h. Band intensities were standardized against β-tubulin and the relative density was analyzed on a Molecular Imager ChemiDoc XRS System (Bio-Rad Laboratories, Hercules, CA, USA) using enhanced chemiluminescence reagent (Thermo Fisher Scientific, Waltham, MA, USA).

### RNA isolation and RT-PCR analysis

Total RNA was isolated from human PBMCs, NRK-52E cells and renal tissues of UAN model rats using TRIzol Reagent (Invitrogen). RNA (1 μg) was used for cDNA synthesis with TransScript Reverse Transcriptase (TransGen) under 42 °C for 30 min, 85 °C, 5 s conditions. Quantitative PCR was performed to measure RNA levels of target genes by using an Icycler IQ Multicolor Real-Time Detection System (Bio-RAD, USA) accordimng to the manufacturer’s instruction of the SYBR Prime Script RT-PCR Kit (Invitrogen). PCR primers were listed as follows: Rat, (IL-6 forward: 5′-GTTCTCTGGGAAATCGTGGA-3′, reverse: 5′-TGTACTCCAGGTAGCTA 3′; IL-8 forward: 5′-TCTGTGTGGATTGGTGGCTCT-3′, reverse 5′-GACTCATCGTACTCCTGCTTGCT-3′; ANRIL forward: 5′-TTATGCTTTGCAGCACACTGG-3′, reverse 5′-GTTCTGCCACAGCTTTGATCT-3′; GAPDH forward: 5′-ACAGCAACAGGGTGGTGGAC-3′, reverse: 5′-TTTGAGGGTGCAGCGAACTT′); Human, (ANRIL forward: 5′-GCCTCATTCTGATTCAACA-3′, reverse: 5′-TAGAAAGCAGTACTGACTCGG-3′; IL6 forward: 5´-ACTCACCTCTTCAGAACGAATTG-3′, reverse: 5′-CCATCTTTGGAAGGTTCAGGTTG-3′; IL8 forward: 5′-CACTGTGTGTAAACATGACTTC-3′, reverse: 5′-ATGCACTGACATCTAAGTTCTT-3′; GAPDH forward: 5′-GCTCTCTGCTCCTCCTGTTC-3′, reverse: 5′-ACGACCAAATCCGTTGACTC-3′). The PCR conditions were as follows: initial denaturation at 95 °C for 10 min; 40 cycles of 95 °C for 15 s and 60 °C for 60 s. The relative gene expression was calculated using the comparative CT Method. The samples were normalized to the GAPDH mRNA level.

### Histopathology

Separated bilateral kidneys from rats in each group were fixed in 4% paraformaldehyde, dehydrated with a graded series of ethanol, infiltrated with xylene, and then embedded in paraffin. The sections (5 μm) were stained with hematoxylin and eosin (HE) staining and Masson’s trichrome staining (all from Sigma; St. Louis, MO) following the manufacturer’s instruction, Ki67 (Zhongshan Goldenbridge Biotechnology, Beijing, China) staining was used to evaluate the kidney cell proliferation.

### TUNEL staining

TUNEL was performed on paraffin sections of kidney tissue from rats by using commercial kits and 3,3′-diaminobenzidine (DAB) staining kits (Roche, Shanghai, China) to detect kidney apoptosis in accordance with the instructions of the manufacturer. TUNEL-positive cells (brown) were observed under a microscope (Olympus, Tokyo, Japan).

### Statistical analysis

Data were expressed as mean ± standard deviation (SD). Statistical analyses were performed by SPSS 18.0 software (IBM, Armonk, NY, USA). Significant differences was evaluated using an unpaired two-tailed Student’s *t* test or by one-way analysis of variance (ANOVA), followed by the *Dunnett’s* or *Tukey’s* test. *P* < 0.05 was considered statistically significant.

## Results

### ANRIL and inflammatory factors were highly expressed in patient with UAN

**Fig. 1:**
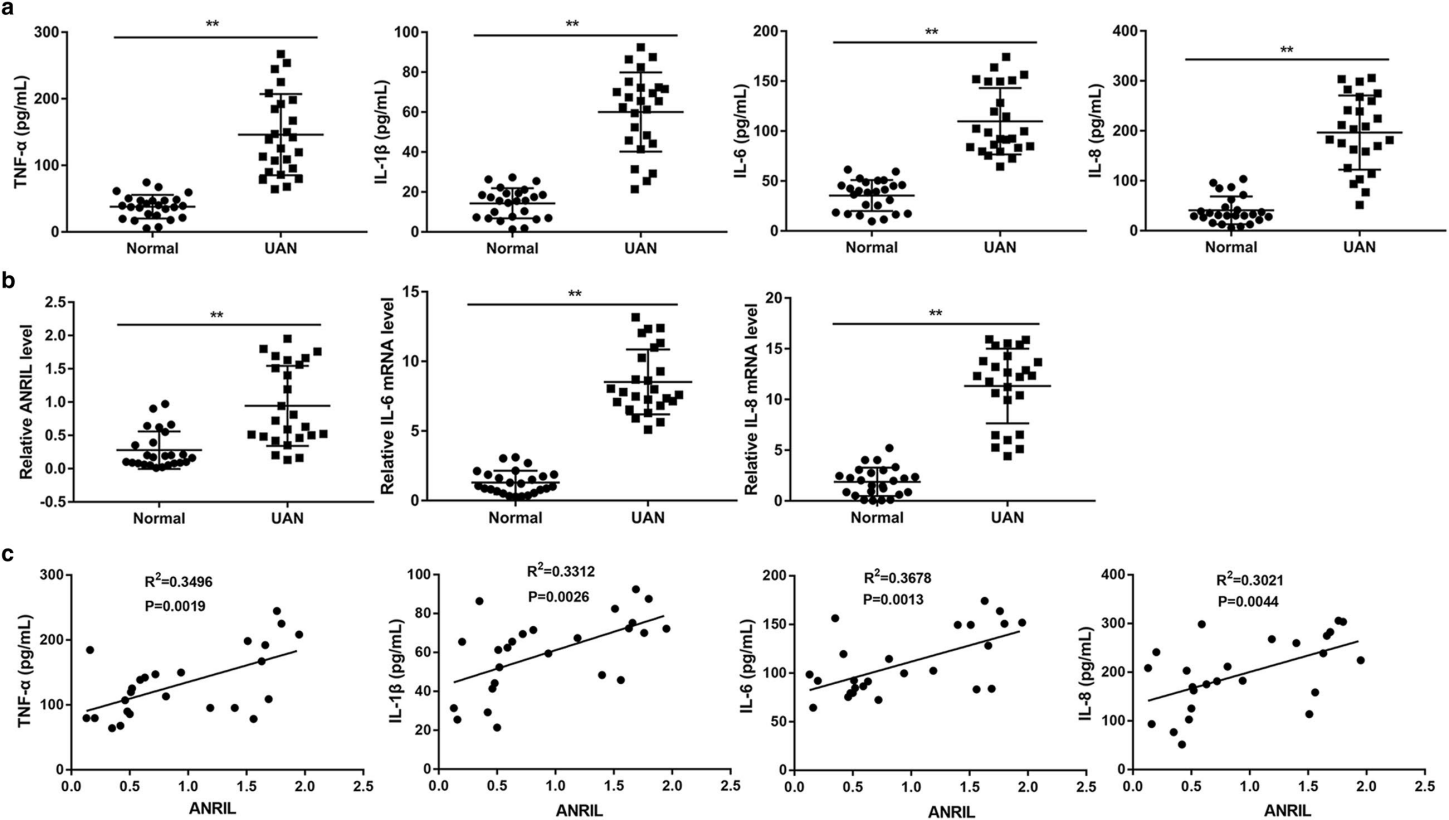
Expression of ANRIL and inflammatory factors in patient with UAN (n = 25) and healthy volunteers (n = 25). **a** ELISA analysis of inflammatory factor expression. **P < 0.01 vs. control. **b** QRT-PCR analysis of inflammatory factor and ANRIL expression. **P < 0.01 vs. control. **c** There was a positive correlation between ANRIL expression and the level of TNF-α, IL-1β, IL-6, and IL-8. **P < 0.01

Serum inflammatory factors in patient with UAN were detected using ELISA. The results showed that TNF-α, IL-1β, IL-6 and IL-8 level was significantly upregulated in patient with UAN compared to that in normal control (*P* < 0.01, Fig. 1a). In addition, qRT-PCR was performed to analyze the expression of ANRIL, IL-6 and IL-8 mRNA in PBMCs from patient with UAN and normal control. As shown in Fig. 1b, ANRIL, IL-6 and IL-8 mRNA levels in patient with UAN were observable raised than control (*P* < 0.01). Furthermore, there was a positive correlation between ANRIL expression and the level of correlated inflammatory factors in the serum from patients (*P* < 0.01, Fig. 1c).

### The role of rhein on renal protection in hyperuricemic rat

**Fig. 2:**
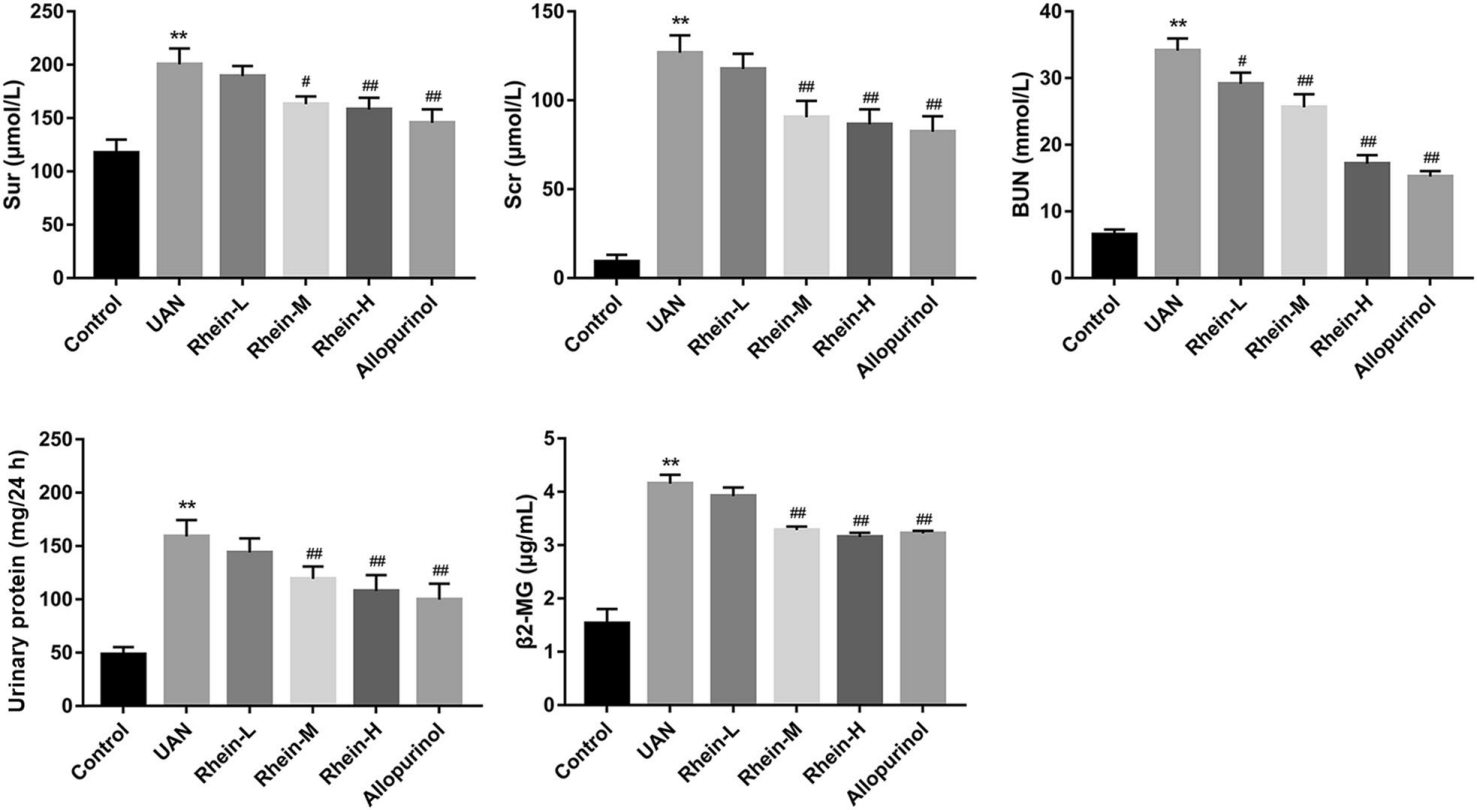
Effects of rhein and allopurinol on renal dysfunction induced by adenine in rats. Parameters of renal dysfunction: serum uric acid (Sur), serum creatinine (Scr), blood urea nitrogen (BUN), 24 h-proteinuria and serum β2-microglobulin (β2-MG) level. **P < 0.01 vs. control; ^#^*P* < 0.05, ^##^*P* < 0.01 vs. UAN group

Levels of Sur, Scr, BUN, 24 h-proteinuria and serum β2-MG in adenine-induced rat model of adenine-induced hyperuricemia were significantly increased compared with that in the control group (*P* < 0.01, Fig. 2), indicating that the UAN model rats were made successfully. In hyperuricemic mice, treatment of rhein at 150, 300 mg/kg or allopurinol at 10 mg/kg availably decreased the Sur (*P* < 0.05, *P* < 0.01, and *P* < 0.01), Scr (*P* < 0. 01, *P* < 0. 01, and *P* < 0. 01) and BUN (*P* < 0. 01, *P* < 0. 01, and *P* < 0. 01), 24 h-proteinuria (*P* < 0. 01, *P* < 0. 01, and *P* < 0. 01) and serum β2-MG (*P* < 0. 01, *P* < 0. 01, and *P* < 0. 01) levels (Fig. 2).

**Fig. 3:**
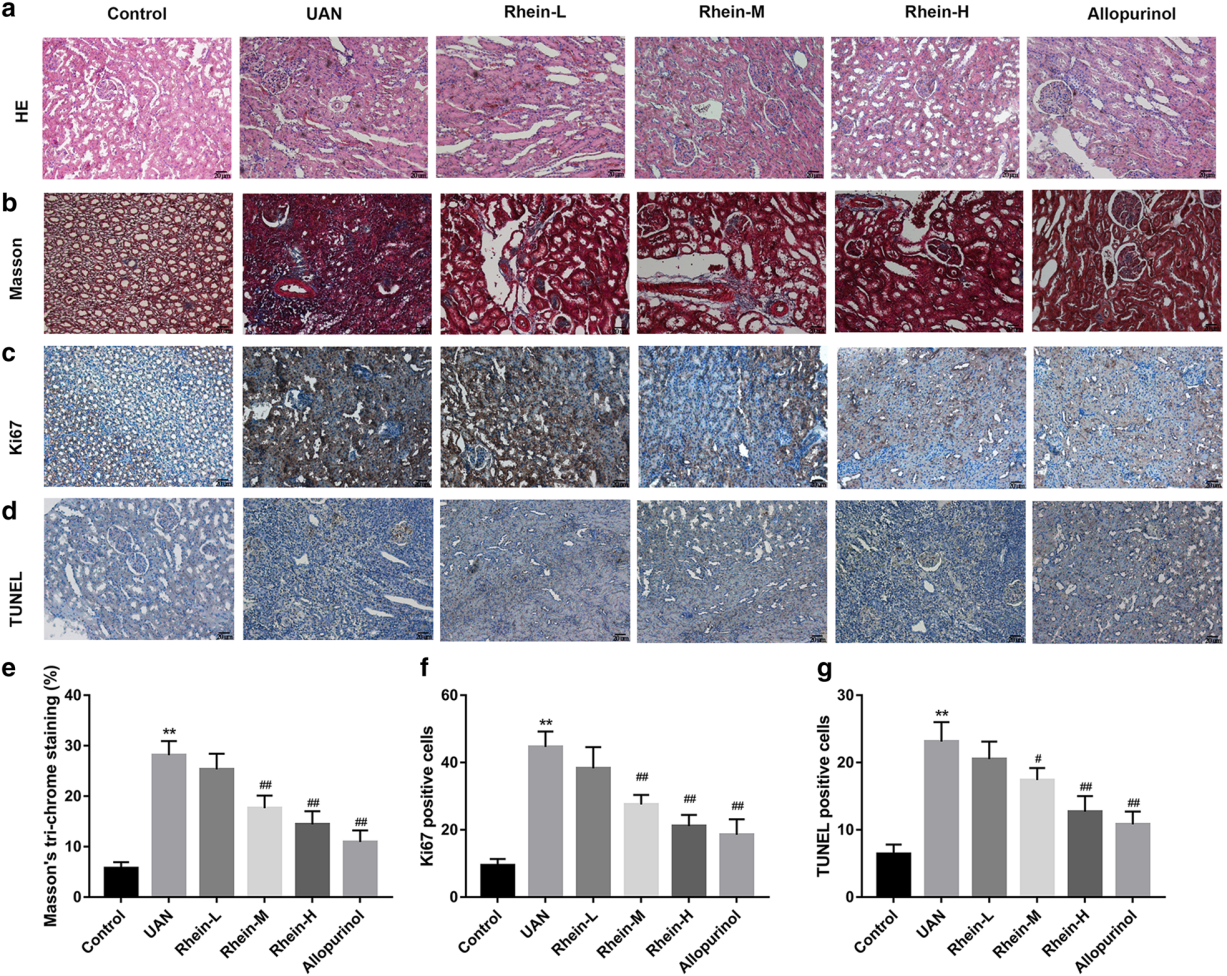
Protective effects of rhein and allopurinol on adenine-induced UAN in rats. **a** The representative pictures of HE staining. Scale bar = 20 μm. **b** Representative pictures of Masson trichrome staining. Scale bar = 20 μm. **c** Representative pictures of immunohistochemical of Ki-67. Scale bar = 20 μm. **d** Representative pictures of TUNEL staining. Scale bar = 20 μm. **e** Quantitative analysis of collagen deposition and fibrosis. **f** Quantitative analysis of Ki-67 staining. **g** Quantitative analysis of TUNEL-positive cells number. **P < 0.01 vs. control; ^#^*P* < 0.05, ^##^*P* < 0.01 vs. UAN group

In addition, HE and Masson trichrome staining of renal tissue from adenine-treated rat showed glomeruli partial atrophy, tubular cells diffuse swelling in a different degree, deposition of sodium urate crystals in the tubules and interstitium and proliferation of fibrous tissue, but the treatment of 150, 300 mg/kg rhein or 10 mg/kg allopurinol attenuated kidney damage with various extents (*P* < 0.01, Fig. 3a, b and e). Furthermore, immunohistochemical analysis showed that the expression of renal Ki-67 and TUNEL-positive cells was markedly increased in the model group (*P* < 0.01, Fig. 3c and f, d and g). However, the remarkable reduction in Ki-67 and TUNEL-positive cells was observed in the rhein or allopurinol treated group as compared to that in the model group (*P* < 0.05, Fig. 3c and f, d and g).

### Rhein inhibited expressions of inflammation cytokines and ANRIL

**Fig. 4:**
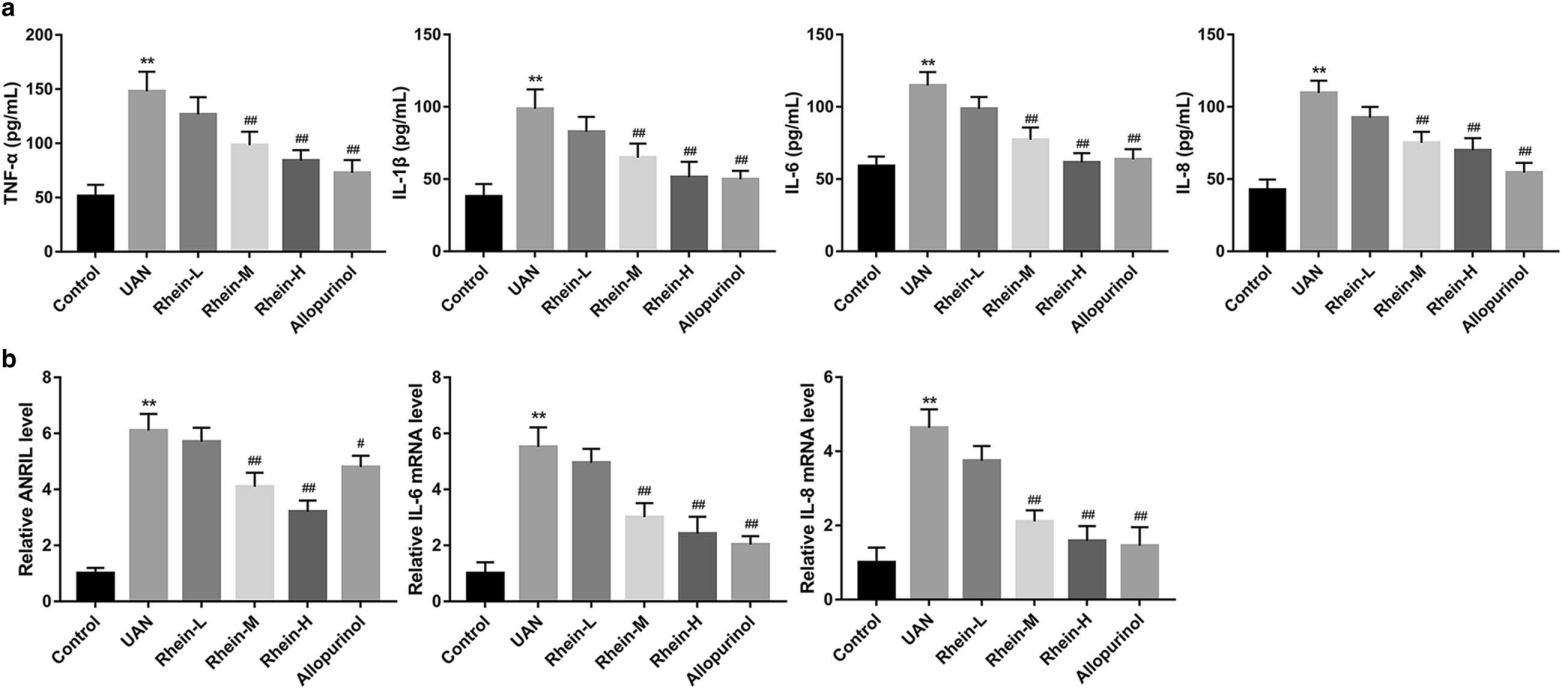
Effects of rhein on levels of inflammation factors and ANRIL in UAN rats. **a** ELISA analysis of TNF-α, IL-1β, IL-6 and IL-8 in serum. **b** qRT-PCR analysis of ANRIL, IL-6 and IL-8 in the kidneys of UAN rats. **P < 0.01 vs. control; ^##^*P* < 0.01 vs. UAN group

Serum inflammatory factors were determined in renal by using ELISA. Levels of TNF-α, IL-1β, IL-6 and IL-8 in the model group were markedly elevated than that in control (*P* < 0.01, Fig. 4a), but treatment of rhein at 150, 300 mg/kg doses and allopurinol significantly decreased the TNF-α, IL-1β, IL-6 and IL-8 levels in the UAN rats (*P* < 0.01, Fig. 4a). Compared with the control group, adenine-induced rat model group in ANRIL, IL-6 and IL-8 level had a marked increase as demonstrated by qRT-PCR (*P* < 0.01, Fig. 4b). However, in the UAN rats, rhein at 150, 300 mg/kg and allopurinol effectively suppressed ANRIL, IL-6 and IL-8 expression (*P* < 0.01, Fig. 4b).

### ANRIL-mediated inflammatory response inhibited protective effect of rhein

**Fig. 5:**
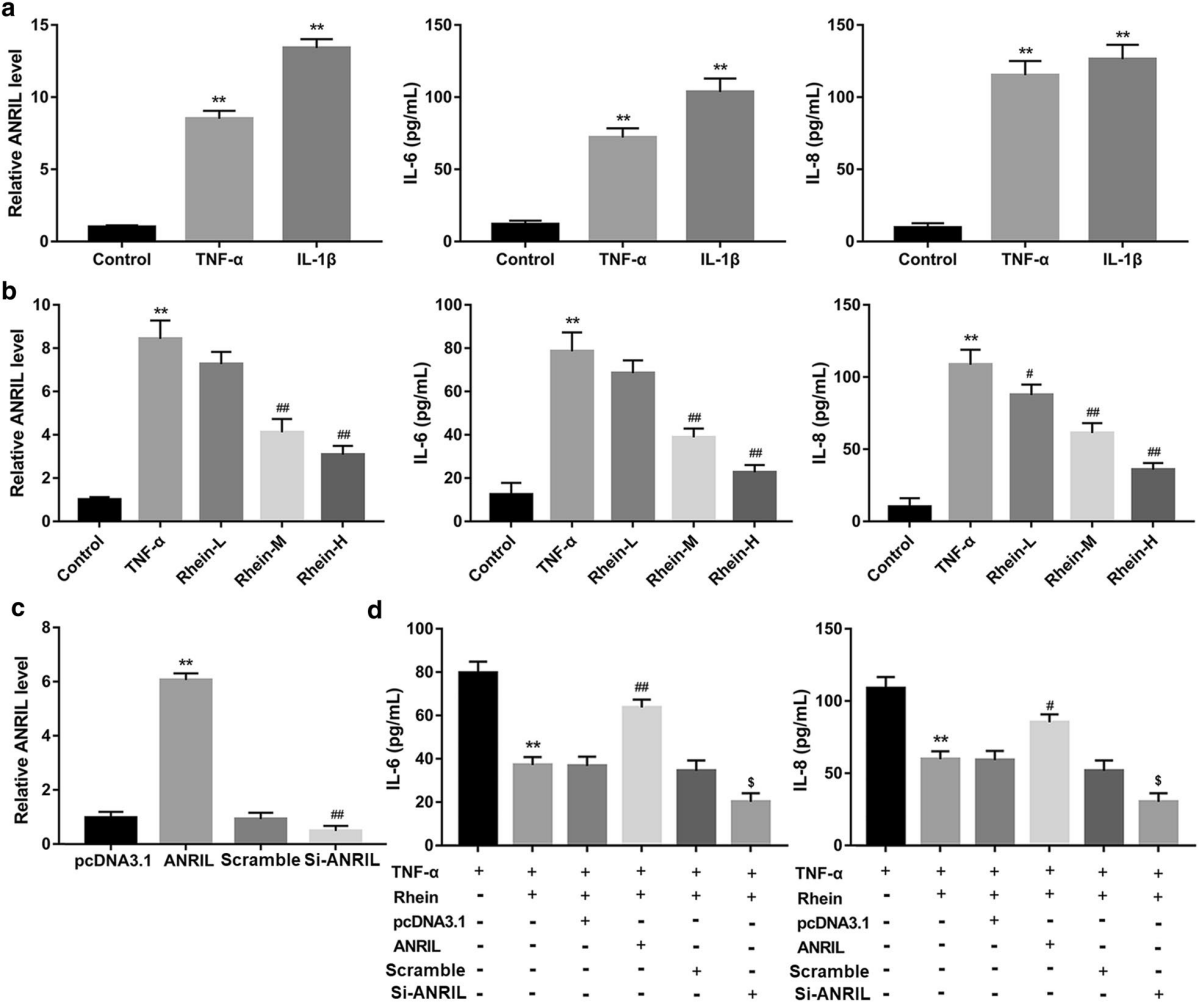
ANRIL effect on treatment of rhein in NRK-52E cells. qRT-PCR and ELISA analysis of ANRIL, IL-6 and IL-8. **a** TNF-α-induced ANRIL, IL-6 and IL-8 expression, **P < 0.01 vs. control. **b** Rhein suppressed TNF-α-induced ANRIL, IL-6 and IL-8 expression. **P < 0.01 vs. control; ^#^*P* < 0.05, ^##^*P* < 0.01 vs. UAN group. **c** The transfection efficiency of ANRIL-overexpression and interference plasmid. **d** ANRIL suppressed anti-inflammatory effects of rhein. **P < 0.01 vs. TNF-α treatment group; ^#^*P* < 0.05, ^##^*P* < 0.01 vs. pcDNA3.1 group. ^$^*P* < 0.05 vs. scramble groups

It has been reported that ANRIL is a novel target of NF-κB signaling [22]. We wondered whether ANRIL effects on inflammatory response to involve in treatment effect of rhein on UAN. A TNF-α-induced NRK-52E cells model was used to probe the function and mechanism of ANRIL in anti-inflammatory effect of rhein. We first examined ANRIL mRNA level and supernatant pro-inflammatory factor level in NRK-52 E cells stimulated with NF-κB activators including TNF-α and IL-1β by qRT-PCR and ELISA. As shown in Fig. 5a, TNF-α and IL-1β induce ANRIL, IL-6 and IL-8 high expression (*P* < 0.01). Furthermore, rhein at 20 and 40 μg/mL doses was found to dramatically suppress expression of ANRIL, IL-6 and IL-8 induced by TNF-α (*P* < 0.01, Fig. 5b). To verify whether ANRIL-mediated inflammation is associated with treatment of rhein in UAN, we first transfected plasmid which carries ANRIL gene or si-ANRIL into NRK-52E cells. The results showed that ANRIL gene was up-expressed in the ANRIL overexpression group, down-expressed in RNA interference group than that in their control group as demonstrated by qRT-PCR (Fig. 5c), reflecting transfection cells with the recombinant plasmid successfully expressed or interfere ANRIL gene. In addition, over-expression of ANRIL significantly increased the IL-6 and IL-8 levels (Additional file 1: Fig. S1A). But, 20 μg/mL rhein markedly reduced TNF-α-induced IL-6 and IL-8 expression (Fig. 5d, *P* < 0.01 vs. control). However, this effect of rhein was abolished by upregulation of ANRIL (Fig. 5d and Additional file 1: Fig. S1A, *P* < 0.05 vs. vector plasmid group), whereas enhanced by downregulation of ANRIL (Fig. 5d and Additional file 1: Fig. S1C, *P* < 0.05 vs. scramble group). ANRIL had no effect on p-p65 expression (Fig. 5d and Additional file 1: Fig. S1B and D).

## Discussion

It has been known for many years that patients with hyperuricemia may develop severe kidney disease, in which intrarenal urate crystal deposition is prominent [23, 24]. Pathogenesis is mainly due to serum urate levels at concentrations above the solubility point of uric acid, resulting in an intensive inflammatory response [25]. Additionally, ANRIL, a well-known functional lncRNA, plays a critical role in multiple human diseases. It is proposed that ANRIL is involved in inflammatory responses in coronary artery disease [22]. In our study, we found that under pathological conditions, serum inflammatory factor levels were significantly upregulated in patient with UAN than that in normal control. Besides, qRT-PCR data of ANRIL, IL-6 and IL-8 in patients with UAN showed an observable rise than control. These results revealed that inflammatory factor and ANRIL were correlated with UAN.

Anti-inflammatory, antioxidative, antitumor and nephroprotective effect of rhein has been reported in many studies [26–28]. It has been showed that rhein is the central mediator of inflammatory processes linked to nephroprotective effects in hyperuricemic mice [5]. In the current study, rhein dramatically reduced levels of Sur, Scr, BUN, 24 h-proteinuria and β2-MG in adenine-induced rat. In addition, histology of renal tissue showed that different concentrations of rhein respectively attenuated kidney damage with various extents. Meanwhile, immunohistochemical analysis showed that the expression of renal Ki-67 and TUNEL-positive cells was markedly increased in the model group, but rhein treatment reduced the number of Ki-67 and TUNEL-positive cells. Furthermore, rhein significantly decreased levels of TNF-α, IL-1β, IL-6 and IL-8 in the UAN rats. These data indicate that rhein has a considerable effect on nephroprotective agent and hyperuricemia-induced inflammatory response in UAN rats.

LncRNAs are newly-developing regulators, involved in modulating human immune response [29, 30]. Correspondingly, increasing evidence suggests that the inflammation plays an important role in response to deposition of uric acid resulting from pathologic damage [31, 32]. NF-κB is a key moderator of inflammation signaling by regulating the expression of a variety of cytokines and chemokines [33]. Study of uric acid could activate NF-κB signaling has been demonstrated in rat renal tubular epithelial cells [34]. Of note, research indicates that ANRIL is involved in TNF-α-induced NF-κB signaling pathway to mediated inflammatory response in endothelial cells [22]. In our study, we found that TNF-α and IL-1β induce ANRIL, IL-6 and IL-8 high expression. However, rhein dramatically suppresses expression of ANRIL, IL-6 and IL-8 induced by TNF-α. Furthermore, rhein markedly reduced TNF-α-induced IL-6 and IL-8 expression, but this effect of rhein was abolished by upregulation of ANRIL. These data indicate that the ANRIL is involved in protective effect of rhein by regulating inflammatory cytokines.

## Conclusions

In conclusion, our data demonstrate that ANRIL is associated with inflammatory response in UAN. Besides, rhein showed an effect of anti-inflammatory and renal protection in hyperuricemic model mice. ANRIL-mediated inflammatory response suppresses protective effect of rhein. ANRIL as a modulator of inflammatory response for the prevention and treatment of UAN has been proposed, which has an important theoretical significance and potential application value.

## Additional file

**Additional file 1: Fig. S1.** ANRIL effect on inflammation response in NRK-52E cells. (A)The effect of ANRIL overexpression on TNF-α-induced IL-6 and IL-8, **P < 0.01 vs. TNF-α, ^##^*P* < 0.01 vs. TNF-α + pcDNA3.1, ^$$^*P* < 0.01 vs. TNF-α + rhein + pcDNA3.1. (B) Overexpression of ANRIL had no effect on p-p65 expression. **P < 0.01 vs. TNF-α. (C) The effect of ANRIL interference on TNF-α-induced IL-6 and IL-8. **P < 0.01 vs. TNF-α, ^##^*P* < 0.01 vs. TNF-α + scramble, ^$$^*P* < 0.01 vs. TNF-α + rhein + scramble. (D) Interference of ANRIL had no effect on p-p65 expression. **P < 0.01 vs. TNF-α.

## Abbreviations

BUN: blood urea nitrogen
ELISA: enzyme-linked immunosorbent assay
LncRNAs: long non-coding RNAs
PBMCs: peripheral blood mononuclear cells
Scr: serum creatinine
Sur: serum uric acid
UAN: uric acid nephropathy
