# Supplementary figures and images for: Long non-coding RNA ANRIL-mediated inflammation response is involved in protective effect of rhein in uric acid nephropathy rats
Source: Cell Biosci. 2019 Jan 17;9:11. doi: 10.1186/s13578-019-0273-3 (PMC6335822; doi:10.1186/s13578-019-0273-3)

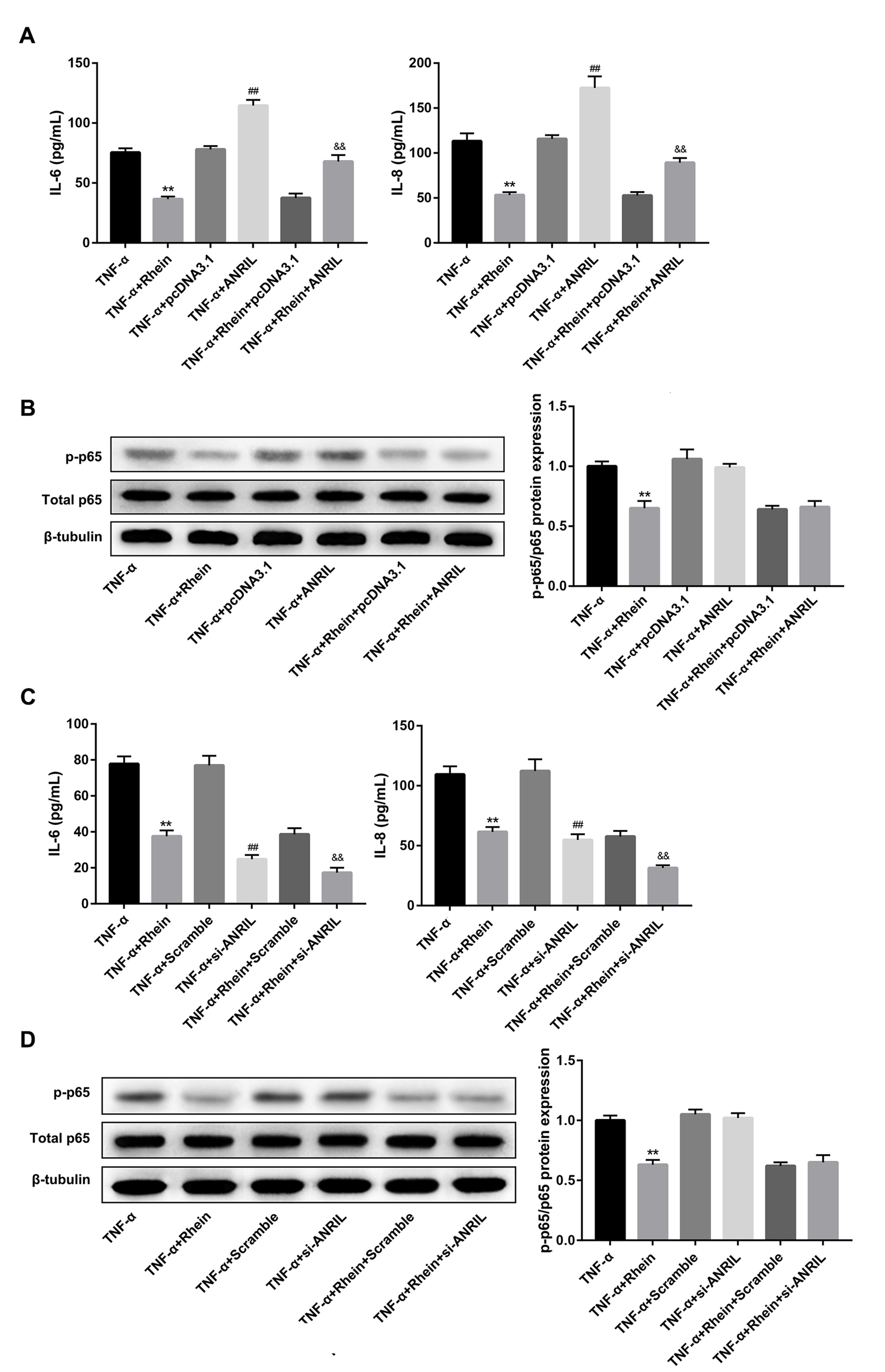

Supplement: Supplementary file 1 — Additional file 1: Fig. S1. ANRIL effect on inflammation response in NRK-52E cells. (A)The effect of ANRIL overexpression on TNF-α-induced IL-6 and IL-8, **P < 0.01 vs. TNF-α, ##P < 0.01 vs. TNF-α + pcDNA3.1, $$P < 0.01 vs. TNF-α + rhein + pcDNA3.1. (B) Overexpression of ANRIL had no effect on p-p65 expression. **P < 0.01 vs. TNF-α. (C) The effect of ANRIL interference on TNF-α-induced IL-6 and IL-8. **P < 0.01 vs. TNF-α, ##P < 0.01 vs. TNF-α + scramble, $$P < 0.01 vs. TNF-α + rhein + scramble. (D) Interference of ANRIL had no effect on p-p65 expression. **P < 0.01 vs. TNF-α. [file 13578_2019_273_MOESM1_ESM.tif]
